# Results of Clinicians Using a Therapeutic Robotic System in an Inpatient Stroke Rehabilitation Unit

**DOI:** 10.1186/1743-0003-8-50

**Published:** 2011-08-26

**Authors:** Hussein A Abdullah, Cole Tarry, Cynthia Lambert, Susan Barreca, Brian O Allen

**Affiliations:** 1 School of Engineering, University of Guelph, Guelph, N1G 2W1, Ontario, Canada; 2 Hamilton Health Sciences, 237 Barton Street West, Hamilton, L8L 2 × 2, Ontario, Canada; 3 School of Rehabilitation Science, McMaster University, Hamilton, L8S 1C7, Ontario, Canada; 4 Department of Mathematics and Statistics, University of Guelph, Guelph, N1G 2W1, Ontario, Canada

## Abstract

**Background:**

Physical rehabilitation is an area where robotics could contribute significantly to improved motor return for individuals following a stroke. This paper presents the results of a preliminary randomized controlled trial (RCT) of a robot system used in the rehabilitation of the paretic arm following a stroke.

**Methods:**

The study's objectives were to explore the efficacy of this new type of robotic therapy as compared to standard physiotherapy treatment in treating the post-stroke arm; to evaluate client satisfaction with the proposed robotic system; and to provide data for sample size calculations for a proposed larger multicenter RCT. Twenty clients admitted to an inpatient stroke rehabilitation unit were randomly allocated to one of two groups, an experimental (robotic arm therapy) group or a control group (conventional therapy). An occupational therapist blinded to patient allocation administered two reliable measures, the Chedoke Arm and Hand Activity Inventory (CAHAI-7) and the Chedoke McMaster Stroke Assessment of the Arm and Hand (CMSA) at admission and discharge. For both groups, at admission, the CMSA motor impairment stage of the affected arm was between 1 and 3.

**Results:**

Data were compared to determine the effectiveness of robot-assisted versus conventional therapy treatments. At the functional level, both groups performed well, with improvement in scores on the CAHAI-7 showing clinical and statistical significance. The CAHAI-7 (range7-49) is a measure of motor performance using functional items. Individuals in the robotic therapy group, on average, improved by 62% (95% CI: 26% to 107%) while those in the conventional therapy group changed by 30% (95% CI: 4% to 61%). Although performance on this measure is influenced by hand recovery, our results showed that both groups had similar stages of motor impairment in the hand. Furthermore, the degree of shoulder pain, as measured by the CMSA pain inventory scale, did not worsen for either group over the course of treatment.

**Conclusion:**

Our findings indicated that robotic arm therapy alone, without additional physical therapy interventions tailored to the paretic arm, was as effective as standard physiotherapy treatment for all responses and more effective than conventional treatment for the CMSA Arm (p = 0.04) and Hand (p = 0.04). At the functional level, both groups performed equally well.

## Introduction

Stroke remains an international leading cause of partial or full loss of motor function, with about 75% of individuals failing to regain functional use of their paretic arm and hand [1,2]. There is increasing evidence that enriched, functional, active, and repetitive practice of movement may have a profound effect on recovering impaired motor function in sub-populations after a stroke or an acquired brain injury [3,4]. Animal studies have shown that, not only practice, but also novel and varied activities for the upper limb, appear to enhance synaptic elaboration [5,6]. The Canadian Best Practice Recommendations advise repetitive and intense use of novel tasks to challenge individuals to acquire the necessary motor skills [7]. Passive [8,9] and active assistance upper limb movements [10,11] seem to improve recovery by their effect on somatosensory input, motor planning, soft tissue properties, and spasticity. However, these intensive approaches are often slow, lengthy, and costly, as they usually require a clinician to work individually with patients. A lack of resources may result in rehabilitation treatment being inadequate in its duration and intensity, thereby not optimizing functional return [12]. With the incidence of stroke expected to rise exponentially within the next 20 years [7], demand for therapy is also expected to increase. As health care resources are limited, technology has the potential to play a supportive role in rehabilitation. Therefore, it is timely to investigate more cost effective intelligent systems that use novel and varied computer programming to offer additional practice opportunities.

Physical rehabilitation is an area where robotics could contribute significantly to improved motor return for individuals following a stroke [13-15]. A robotic system with controllable movement velocity and supported by intelligent sensing capability may be a vital assistant in today's physical therapy centres. Individuals' data may be objectively recorded, helping therapists and physicians monitor and evaluate the patient's progress and the treatment intervention.

### Rehabilitation Robotics

During the 1990s, there was a growing interest in the field of therapeutic applications of robots [16]. One of the first contributions to robots in physiotherapy was conducted at the Rehabilitation Institute of Michigan [17]. The device was programmed to do five simple movement patterns, each consisting of eight points. Although the literature reports on many robot-assisted devices designed to deliver therapy for the paretic upper limb, only seven have been tested in clinical trials (MIT-Manus [18-20], MIME [21,22], ARM-Guide [23], GENTLE [24], Bi-Manu-Track [25], BATRAC [26] and REHA-ROB [27]). Six of the trials reported above used the Fugl Meyer assessment scale while the ARM trial used the Chedoke McMaster Stroke Assessment (CMSA). Sivan at el. have classified and evaluated the outcome measures currently used in robot-assisted exercise trials (RAET) in stroke through a systematic review published in [28]. They have found that "Chedoke Arm and Hand Activity Inventory (CAHAI) scale would seem to be an appropriate activity scale, but has not been used in RAET" [28].

MIT-Manus is a planar module that provides 2 translation DOFs (degrees of freedom) for elbow and forearm motion and repetitive massed practice of reaching toward an end point using impedance control [15]. An evaluation of this therapeutic robot has been reported in several studies for treatment of acute and chronic post-stroke hemiparesis [15 (n = 20), 18 (n = 76), and 19 (n = 47)]. The results showed that the robotic therapy group had significantly more improvement in motor function; however, Brewer (2007) [29] concluded "these results are not definitive because (a) the control (conventional therapy) group had significantly poorer motor and cognitive function at baseline, and (b) the treatment group received 4-5 hours of traditional therapy compared with 30 minutes for the control group". The MIME (Mirror-Image Motion Enabler) incorporated an industrial robot manipulator (Puma 560) that applied forces to the paretic forearm in 3-dimensional space [30], where the non-affected arm guided the paretic arm. The MIME used uni-manual and bi-manual active and passive arm exercises and was directly compared to conventional neurodevelopmental treatment (n = 27). The MIME treatment group had greater increases in strength and reaching. However, there was little difference between the two groups at 6 months follow up [29].

The Rehabilitation Institute of Chicago built the ARM Guide (Assisted Rehabilitation and Measurement) that has 3-controlled DOF to provide assistive therapy to patients' upper extremity with chronic hemiparesis [23]. The ARM assistive therapy compared directly with conventional therapy (n = 19) showed no difference between the two methods [30]. The GENTLE's project utilized a 3 DOF haptic interface and Virtual Reality (VR) technologies to enhance patient attention and motivation [31]. In this clinical trial (n = 20), 10 subjects used the robot reaching therapy and another 10 used the sling suspension phase. The trend emerging from the results showed that the robotic system had a more positive treatment effect than single plane repetitive exercises; however, the results lacked statistical significance and further research in the form of RCT was warranted. Another system, the Bi-Manu-Track robot, enabled basic bilateral passive and active practise of two movements, forearm pro-supination and wrist flexion and extension in a mirror-like or a parallel fashion [25]. In a clinical trial (n = 44) acute stroke patients received assistive or resistive force robotic therapy. The patients improved and maintained their advantage three months after treatment compared to a control group who received movement practice aided by electrical stimulation [25]. The BATRAC is an apparatus comprised of 2 independent T-bar handles that can be moved by the patient's hands (through shoulder and elbow flexion/extension) on a horizontal plane. Nineteen patients with chronic hemiparetic stroke used the system in a single design group for 6-9 weeks. The patients showed improvement in several key measures of sensorimotor impairments, with these improvements maintained two months after patients stopped training [26]. The REHAROB therapeutic system uses 2 unmodified ABB industrial robots. The robots are connected to the patient's upper arm and forearm through instrumented orthoses [27]. Thirty patients with hemiparesis were divided randomly into two groups, robot and control. Subjects received 30 minutes of conventional therapy for 20 consecutive work days while subjects in the robotic group received an additional 30 minutes of robot-assisted therapy. This trial concluded that it is useful to patients to supplement conventional therapy with robot assisted therapy.

Nevertheless, none of these studies showed functional improvement. It can be concluded, "a major challenge for related technological developments is to provide engaging patient-tailored task oriented arm-hand training in natural environments with patient-tailored feedback to support learning of motor skills." [32]. Mehrholz et al. reviewed these trials [33], to assess the effectiveness of robot-assisted arm training for improving activities of daily living, arm function and motor strength of patients after stroke. They concluded that although robot-assisted arm therapy may improve impaired motor function and strength of the paretic arm, it did not improve the ability of individuals to perform activities of daily living post-stroke [33]. Therefore, more clinical trials are required to determine whether robotic therapy is feasible in routine stroke rehabilitation. Moreover, these trials would enhance multi-disciplinary team work between the robot developers, therapists, and patients to modify and design more clinically effective devices.

The School of Engineering at the University of Guelph, in consultation with experienced therapists and physiatrists, developed a user-friendly intelligent therapeutic robotic system to provide assistive therapy to patients' upper limbs after stroke. Its design is unique in that the robotic arm is not only bio-sensing driven, but also functions in multiple planes including rotational components. The safety features allow individuals to work in these multidimensional planes with the robotic arm automatically stopping when clients move their limb beyond physiotherapist established fields. The workspace of the exercises is limited in the software to ensure that the patient and robot stay within a reasonable comfort zone. Initial testing and validation of the biomechanical model has been done in the normal population (ages, 18-56; 5 females, 15 males; height, range 1.58 m-1.86 m and weight, range 49 kg-89 kg) [34].

This paper describes the results of a pilot randomized controlled trial (RCT) conducted at Hamilton Health Sciences, Hamilton, Ontario, Canada, to evaluate the clinical utility of our current therapeutic robotic system. Our objectives were to: (i) explore the efficacy of this new type of robotic therapy as compared to standard physiotherapy treatment in treating the post-stroke arm; (ii) evaluate client satisfaction with the proposed robotic systems; and (iii) provide data for the calculation of effect size, power and sample size in anticipation of a large, multi-centered RCT.

## Methods

Ethics approval was obtained from Hamilton Health Sciences and University of Guelph Research Ethics Boards. The Ministry of Health in Canada approved the system as a new class II medical device in Canada for investigational trials. If the individuals met the inclusion criteria and gave informed consent, they were then assessed by an occupational therapist who was blinded to the study and not directly involved with patient care. Individuals were admitted to the Chedoke Stroke Rehabilitation Unit at Hamilton Health Sciences, Hamilton, Ontario.

### Inclusion Criteria

Individuals (i) gave informed consent; (ii) had a diagnosis of a first single, unilateral stroke; (iii) were between the ages of 16-90; (iv) were 2-8 weeks post stroke; (v) had arm motor impairment between stages 1-4 as measured by the CMSA; and (vi) were able to follow simple instructions.

### Exclusion Criteria

Individuals who had (i) shoulder pain between 1-3, as measured by the CMSA pain inventory scale, i.e., severe constant pain and/or (ii) the presence of other pathology in the affected shoulder or elbow.

#### Outcome Measures

The goal of rehabilitation is to increase function and for this reason, we selected the shortened version of the Chedoke Arm & Hand Activity Inventory (CAHAI-7) as the primary outcome measure. This assessment (range 7-49) of upper limb performance using functional items was specifically designed for the stroke population. The CAHAI has been shown to be more sensitive to clinically important change in upper limb function than the gold standard, the Action Research Arm Test [35-39]. The CAHAI assesses both arm and hand stabilization and manipulation abilities using everyday functional items deemed important by individuals who have experienced a stroke. It has excellent psychometric properties and measures how much the paretic upper limb contributes to the completion of everyday functional tasks, an important goal for patients.

Secondary outcome measures were: (i) an impairment measure, the CMSA, which allowed us to stratify motor impairment of the arm and hand into separate ordinal stages (range, 1-7) as the prognostic literature attests to the strong relationship between the degree of motor return and the amount of function gained [39] and (ii) client satisfaction using a 10-point Likert scale (LS), measuring the client's perception of their level of enjoyment and degree of improvement (Table 1).

**Table 1:** Likert scales

**a) **Circle the number that best describes your feelings about the type of therapy you got for your arm and hand										
	**I enjoyed the type of arm and hand treatment**									

	**1**	**2**	**3**	**4**	**5**	**6**	**7**	**8**	**9**	**10**

	**Not at all**									**Very much**

b) Circle the number that best describes how much you feel your arm and hand has gotten better										

	**My arm and hand improved**									

	**1**	**2**	**3**	**4**	**5**	**6**	**7**	**8**	**9**	**10**

	**Not at all**									**Very much**

### Robotic Systems

**Figure 1:**
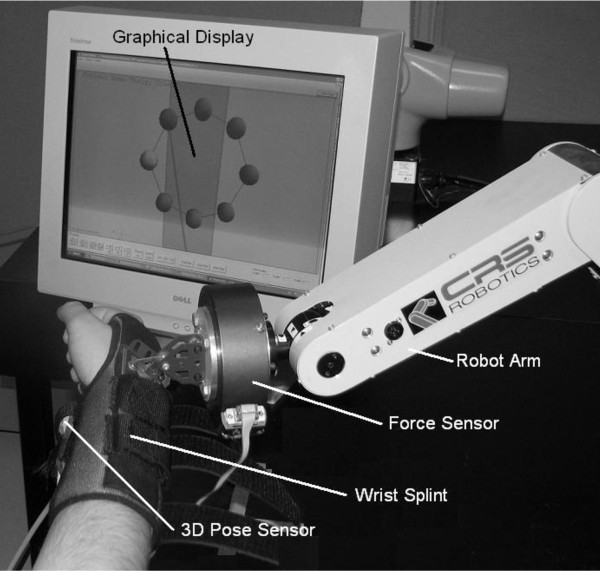
Theraputic robto setup and main components

A novel sensory system and upper limb bio-mechanical model combined with a graphical interface were used to convert an industrial robot (5degrees of freedom (DOF) desktop robot with position based control) into a safe rehabilitation tool for physiotherapy (figure 1). A 6 DOF force sensor was integrated to implement active control modes, safety systems, and data feed-back. To improve the tracking capabilities of the system, two 3D Space sensors were attached at the wrist and elbow to track the movement and orientation of the forearm and the upper arm. A personal computer (PC) was used as the main controller, and all the sensor outputs were linked to the PC through a universal serial bus data acquisition card. Four robotic control modes were developed and implemented in the system to allow different types of therapy (passive, active assisted, active restricted, and active). The 5-DOF industrial robot used in the system is capable of moving the patient's limb through a variety of motion profiles. This allows the system to train movements on the standard horizontal or vertical planes. The capability of doing exercises in a 3D workspace gives the system the ability to simulate a large number of activities of daily living (ADLs). By incorporating force feedback, the patient can actively control the motion of the robot in a "back-driveable" control mode. A real-time representation of the robot's location and the exercise trajectory was displayed to the patient and therapist. The display was useful to help subjects visualize the exercise that they were performing and where they needed to go next to master the exercise. This representation was in real-time and can be used to monitor the orientation of the upper limb. Feedback in audio and text forms was given to patients.

#### Experimental Procedures

A physiotherapist unrelated to the study randomized the participants into one of two groups using a random number table. A research technican from the University of Guelph collected the biomechanical and progress data from the robotic system at baseline and at discharge. An occupational therapist blinded to patient allocation administered the CAHAI-7 and the CMSA at admission and discharge. At discharge, participants completed two Likert Scales (LSs) that asked them to judge how much (a) they enjoyed the type of arm therapy they received (table 1a) and (b) their paretic arm had improved (table 1b).

**Figure 2:**
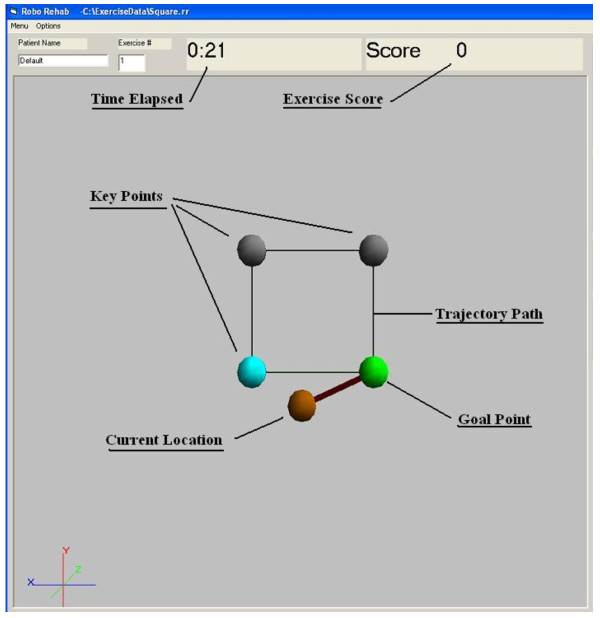
**Square exercise, blue ball is the current location and green ball is the next target location**.

Patients completed a range of exercises that varied in the range of the difficulty, type of task and range of motion. The exercises required the patient to move through a set trajectory that was displayed on a computer screen. Target points were placed along the trajectory as way points for the patient to hit as they move through the exercises. Simple shapes, such as squares (figure 2), triangles, and circles, were used to create the exercise trajectories and were typically performed on a single plane. Clinicians used their clinical judgement to tailor practice sessions and to choose exercises according to the individual's motor and cognitive impairments. A mixture of active, passive, and active assisted control modes were utilized to facilitate motor learning and provide challenge. In active mode, the patient had full control over where the robot moved. This was used with higher level patients capable of making voluntary motions. Passive mode was used to demonstrate the exercises to patients with minimal motor function. In this mode, the robot had full control over its motion and moved the patient through the exercise. Active assisted mode allowed patient control of the robot but the robot would take over control if the patient wasn't progressing through the exercise. The robot would move the patient to the next target point and then relinquish control to the patient again. The exercises consisted of a variety of trajectories to be followed, from tracing simple shapes like squares and circles, to more complex trajectories where a patient would be required to collect a series of objects one at a time and place them in a receptacle.

In addition to the simple trajectory exercises the system had other exercise styles that included object manipulation. Here the patient moved the robot to contact a virtual object (e.g. a cup) on the screen and then placed the object in a new location. The exercises were designed to allow individuals following a stroke to try meaningful functional activities such as reaching for objects, thereby simulating some activities of daily living.

##### A. Experimental (Robotic Therapy) Group

Individuals randomized to the experimental group received 45 minutes supervised training sessions three times a week using only the robot until discharge. No other arm therapy was provided to this group. Clients were seated in a chair or wheelchair in front of the computer screen at a height adjustable table. The trunk was not restrained, but the therapist ensured that the patient was sitting upright, using a pillow if necessary to ensure correct posture. Their affected arm was supported at the wrist by a comfortable forearm splint.

The wrist was in a neutral position with the fingers unsupported. Facing the screen, clients began an exercise program starting with passive movements, progressing to active-assisted, and active as the treating therapist assessed their motor and cognitive ability to interact with the robotic system.

There was a menu of assorted exercises. For example, for an individual with moderately severe cognitive difficulties, the therapist may select movements that use a series of nodes that progressively light up as the person approaches the target. Block practice of the exercise may be more appropriate for this particular individual. For the person whose upper limb is a CMSA Stage 1-2, the therapist may choose a set of active assisted exercises that occur in the typical synergistic flexion and extension patterns. For an individual with no discernible cognitive difficulties, feedback may be random and the practice schedule variable. For a person with a higher level of motor return, i.e., CMSA Stage 3-4, the therapist may choose active exercises occurring both within and outside the typical synergistic patterns (i.e., making a circle, abduction, external rotation). Each exercise was done 10 times for a total treatment time of 45 minutes. An automatic safety feature built into the robotic systems prevented the patient's arm from moving outside the parameters established by the treating therapist. An external stop button was clearly displayed in front of the monitor, which could be manually activated at any time.

##### B. Conventional (Regular Therapy) Group

Similarly, individuals randomized to the conventional group received 45-minutes supervised conventional therapy three times a week until discharge. Assorted techniques for upper extremity retraining were used by the treating therapists (task specific training, passive, active and resistive exercises). Programs progressed as indicated to meet the client's goals.

For both groups, hand exercises were permitted in class settings or with the treating therapist. The amount of occupational therapy where the client practices activities of daily living was recorded.

#### Statistical Analysis

The change in each outcome measure, between baseline and discharge, was statistically analyzed. Because the distribution of CAHAI-7 values was skewed to the right, the difference in the natural log (ln) CAHAI-7 between discharge and baseline was analyzed in order to improve its statistical properties. Thus, for CAHAI-7, results expressed on the original scale are percent changes. The fitted statistical model included the therapy treatment and sex of the patient. The covariates included age of the patient, side of stroke, and the baseline value of the outcome measure. This statistical model was fitted for each outcome measure, using version 9.2 of the software procedure proc glm, from SAS Institute Inc. [40]. Adjusted means were calculated for the therapy treatment and gender of the patient. The significance of the therapy was also assessed, for both conventional and robotic therapy, for the CMSA Arm and Hand, CAHAI-7 and pain experienced.

## Results

Twenty patients in total were admitted to the study. Each subject was assigned to the robotic or conventional therapy group at random, with probability 0.5, independent of previous subject assignments. Hence, the groups may not be exactly equal in size. One subject assigned to the experimental robotic therapy group withdrew before the end of the trial; therefore his data was not included in the analysis, leaving the robotic therapy group with 8 subjects. The results of 19 patients (11 conventional, 8 experimental) are presented. Both groups on average had received 3 therapy sessions a week for 8-11 weeks. Tables 2 and 3 present the demographics of the experimental and control groups, respectively.

**Table 2:** Demographics of the experimental (robotic therapy) group

DEMOGRAPHICS - EXPERMENTAL (Robotic Therapy)						
**Subject**	**Gender**		**Age**	**Weeks Post-CVA**	**Impairment**	**Neglect**
	**Male**	**Female**				**(at admission)**

R1		F	65	4	1	1
R2	M		77	7	2	1
R3	M		74	5	2	2
R4	M		74	4	2	2
R5	M		78	4	2	2
R6		F	77	2	1	2
R7	M		75	2.5	2	1
R8		F	86	6	1	1

**Total**	**5**	**3**				
**Max**.			**86**	**7**		
**Min**.			**65**	**2**		
**Avg**.			**75.7**	**4.3**		

**Impairment**: 1 = Left body (right brain)

2 = Right body (left brain)

3 = Bilateral

**Neglect**: 1 = present, client unable to compensate

2 = present, client able to compensate

3 = absent

**Table 3:** Demographics of the control (conventional therapy) group

DEMOGRAPHICS - CONTROL (Conventional Therapy)						
**Subject**	**Gender**		**Age**	**Weeks Post-CVA**	**Impairment**	**Neglect**
	**Male**	**Female**				**(at admission)**

C1		F	80	1	1	3
C2		F	44	1	2	3
C3	M		66	4	2	3
C4	M		81	4	3	2
C5		F	62	6	1	1
C6		F	82	4	1	2
C7		F	41	3	1	3
C8		F	85	4	1	1
C9	M		75	8	1	3
C10		F	83	7	2	1
C11		F	75	6	2	3

**Total**	**3**	**8**				
**Max**.			**83**	**8**		
**Min**.			**41**	**1**		
**Avg**.			**70.4**	**4.3**		

**Impairment**: 1 = Left body (right brain)

2 = Right body (left brain)

3 = Bilateral

**Neglect**: 1 = present, client unable to compensate

2 = present, client able to compensate

3 = absent

There was no significant difference between the two groups in age. The covariates initial CAHAI and impairment score for the arm and hand and side of stroke were not significant for any outcome measure. Age was a significant covariate for the outcome measures log CAHAI-7 and the survey response level of enjoyment. It was also marginally significant (P < 0.10) for the impairment measure, CMSA for the Arm, and the survey response, degree of improvement.

Data was compared to determine the effectiveness of robot-assisted versus conventional therapy treatments. At the functional level, both groups performed well, with improvement in scores on the CAHAI-7 showing clinical and statistical significance (table 4). Individuals in the robotic therapy group, on average, improved by 62% (7.76 points) while those in the conventional therapy group changed by 30% (2.94 points). Although performance on this measure is influenced by hand recovery, our results showed that both groups had similar stages of motor impairment in the hand (Experimental, mean hand stage at admission 2.63 and at discharge 3.88 vs. Control mean hand stage at admission 2.82 and at discharge 3.27). Furthermore, the degree of shoulder pain, as measured by the CMSA, did not worsen for either group over the course of treatment.

**Table 4:** Mean improvement of CMSA indices under conventional and robot-assisted therapy

	Arm	Hand	Pain	ln(CAHAI-7)	Age
**Conventional therapy (n = 11)**					
Admission	2.36	2.82	5.27	2.27	70.40
Discharge	2.91	3.27	5.55	2.60	
Mean change	0.55	0.45	0.27	0.29^1^	
St. error change	0.28	0.23	0.26	0.10	3.76
P-Value	0.0690	0.0690	0.3000	0.0100	
**Robot-assisted therapy (n = 8)**					
Admission	2.00	2.63	5.25	2.53	75.80
Discharge	3.50	3.88	5.75	2.96	
Mean change	1.50	1.25	0.50	0.48^1^	
St. error change	0.33	0.27	0.30	0.12	4.41
P-value	0.0003	0.0003	0.1130	0.0010	
Difference in treatments	0.95	0.80	0.23	0.20^1^	5.39
Standard error Difference	0.43	0.36	0.39	0.15	5.79
P-value	0.0410	0.0410	0.5710	0.2240	0.3650

^1 ^For ln(CAHAI-7), admission and discharge are the means of the natural log values. However, the mean change and difference in treatments (and their standard errors) are adjusted for age differences of subjects.

For both groups, motor impairment of the affected arm was initially between stages 1-3 (CMSA), with Stage 1 indicating flaccid paralysis, Stage 2 showing beginning of tone with movement being able to be facilitated, whereas Stage 3 indicates that the individual can move their arm in primitive synergistic movements (touch knee, touch chin, shrug shoulders).

Under robot assisted therapy, the CMSA improved significantly for the Arm (1.5, P = 0.0003) and for the Hand (1.25, P = 0.0003). Under the conventional therapy, the CMSA improved, but not significantly for the Arm (0.55, P = 0.069) or for the Hand (0.45, P = 0.069). The improvement of the CMSA of the Arm under robot-assisted therapy was significantly larger than under conventional therapy (P = 0.041) and also for the Hand (P = 0.041) (table 4).

An analysis of power and sample size was conducted [41], based on the error variability seen in this study. This analysis indicates that a trial, with 30 subjects in each group, can detect an improvement in the robotic group, of 0.56 (Arm), 0.46 (Hand), 0.57 (Pain) and 5.6 (CAHAI-7) with 90% power, using a two-tailed test at the 5% level. A trial with 30 subjects in each group will also detect a difference (between the improvement for the robotic assisted therapy and the improvement for conventional therapy) of 0.79 (Arm), 0.65 (Hand), 0.80 (Pain) and 8.0 (CAHAI-7), with 90% power. For the survey outcomes, a trial with 30 subjects per treatment will detect differences of 1.8 (LS-improved), 2.1 (LS-enjoyed treatment) and 4.0 (Hours of active OT & PT/wk).

**Figure 3:**
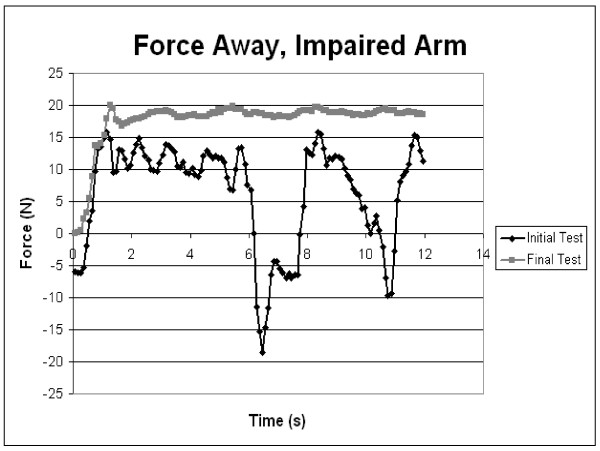
Sample Force test of patient R3 pushing forward (shoulder flexion). Shown are the data from admittance and data on discharge

**Figure 4:**
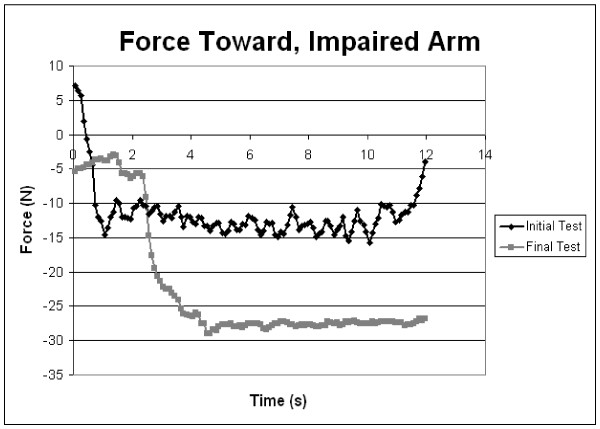
**Sample Force test of patient R3 shoulder extension**. Shown is a comparison of data from admittance and discharge.

**Figure 5:**
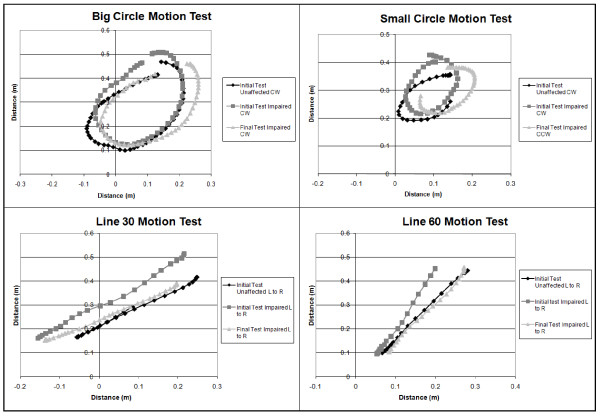
**Sample motion tests showing 4 predefined motions recorded at admission and at discharge for patient R4**. (CW: clock wise, CCW: counter clock wise, L: left, R: Right)

Robotic assessment samples at admission and discharge (figures 3 &4) showed patient [R3]'s force tests while performing shoulder flexion and extension. As the trial progressed, patient R3 displayed better motor control and strength of the upper limb, as illustrated by the smoothness of the lines at discharge compared to the lines at admission. Strength increases were apparent with the increase in the forces applied by the arm over the course of the trial. During the force tests, the patient was not attached to the robot to ensure there was no bias in comparing the two groups. Along with force tests, individuals completed a series of motion tests at admission and discharge. Figure 5 shows the motion tests of patient [R4], comparing movements completed by the unaffected arm to movements completed by the paretic impaired arm at admission and discharge. This example shows how the performance of the impaired arm more closely resembles that of the healthy arm at discharge. These motion tests were intended to compare the degree of control and range of motion that the patient had pre versus post therapy.

**Figure 6:**
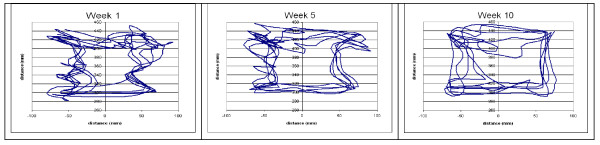
Patient R3 performing a simple square exercise, showing data from admission, part way through treatment and at discharge (direction of movement is clock wise)

Similar data were automatically collected during all exercises performed on the robot. Figure 6 shows data captured over the course of treatment of patient [R3]. This exercise had the person following graphical cues to indicate target locations to trace a square. The robot was in active mode and completely controlled by the person. An average offset was determined for patient [R3] for each of 12 days in which the exercise was administered. The average offset is the average absolute distance from the patient's trace to the target square. This average offset was regressed on the day of testing, measured from the beginning of treatment. The average offset declined by 0.048 per day of treatment (P = 0.076), indicating, for this patient, that the tracings are becoming progressively more square shaped, as treatment progressed.

**Figure 7:**
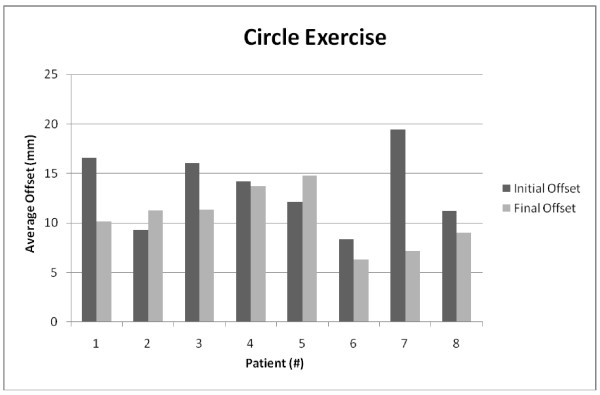
The circle exercise data for the experimental robotic therapy group (8 subjects)

**Figure 8:**
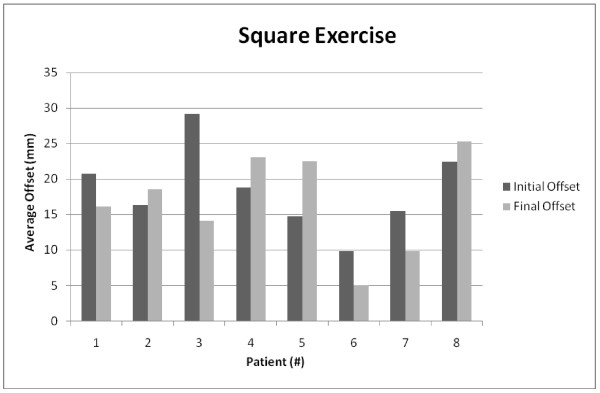
The square exercise data for the experimental robotic therapy group (8 subjects)

To find the trajectory offsets, vectors were created between the target points of the exercise, and the distance from the current trajectory vector to each recorded point was calculated. Figures 7 and 8 compare the average offset of each patient`s first and last attempt at performing the square and circle exercises respectively. Due to the flexibility that the clinicians were allowed in the selection of the exercises and the varying degree of ability of each patient, the exercises were performed at inconsistent difficulty levels and at different volumes. This reduces the ability to compare the data broadly over all the subjects and in some cases on an individual basis.

## Discussion

Although many studies have used the Fugal Myer to measure overall upper limb motor severity following a stroke, we preferred a familiar Canadian measure, the CMSA. This impairment measure divides arm and hand recovery into separate motor stages (1-7), allowing patients to be placed in similar bins as well as allowing researchers the ability to detect whether changes in the arm or hand or in both areas. Gowland (1993) established a high correlation between the CMSA arm and hand impairment to the FM, p = 0.95 [39].

A potential criticism to using a robotic system in an inpatient setting is its clinical utility with an older population, given that the average age of individuals admitted with a stroke is usually in the 65-70 year range and most may be unfamiliar with computer technology. In our study, by chance allocation, the individuals allocated to the experimental group were in fact older, on average, by more than 5 years (mean average age: robotic assisted therapy group, 75.7 (range, 65-86 table 2) vs. conventional group, 70.4 (range, 41-85, table 3.) Nevertheless, these elderly individuals were able to understand and learn how to use the robotic arm. Even though they did not have much experience using computers, they appeared to enjoy this type of treatment as much as the individuals in the control group enjoyed the one-on-one therapist time, as indicated by their LS scores (7.31 for conventional vs. 8.11 for robotic assisted, P = 0.42) (table 5). None indicated that they were intimidated by the robot in any way. Gender was also assessed for statistical significance. It was not found to be statistically significant for the primary outcome measure, CAHAI-7, or for the secondary outcomes CMSA Arm or CMSA Hand. For the LS enjoyment rating scale, gender was significant, where females expressed significantly more enjoyment of their therapy than males (P = 0.026). Males showed a trend towards (P = 0.09) enjoying robotic therapy more than conventional therapy. Women demonstrated equal preference for the two kinds of therapy.

**Table 5:** Likert scale (LS) survey outcomes

	Improved	Enjoyed	Hours
**Conventional Therapy (n = 11)**			
Mean	5.79	7.31	4.09
St. error	0.71	0.65	1.22
**Robotic Therapy (n = 8)**			
Mean	7.35	8.11	6.82
St. error	0.84	0.71	1.44
**Difference in treatments**			

Mean	1.56	0.80	2.73
St. error	1.11	0.97	1.91
P-Value	0.18	0.421	0.171

Reach of the arm is coupled with manipulation of the hand. Improvement in hand function in the robotic group could have resulted from a combination of things: 1) improvements in motor control caused by improvements in the ability to activate spared portions of the damaged corticospinal system; 2) more strength in shoulder and elbow extension, key to reaching for objects in the environment; 3) interaction with simulated real life objects presented on the screen that helped to facilitate hand grip and release; 4) the novelty and challenge of the different type of exercises increased patient motivation, leading to more attention to performance and more cortical activity. As the location and extent of the stroke was not accounted for in our study, and the small sample size, it is difficult to know why there was more improvement in hand control in the robotic group versus the control.

A unique aspect of this study was that there were few restrictions placed on admission criteria. Clients were typical of those admitted to any Canadian inpatient stroke unit, i.e., having multiple combinations of cognitive, physical and somato-perceptual impairments. Most studies using the robotic arm have been done with the chronic stroke population [2,23-25,29,30]. In this study, individuals were still in the subacute stage (weeks post onset to admission to rehabilitation ranged from 1-8 weeks). The literature would appear to support earlier intervention. Feed-back from the therapists and patients will be used for future development of the system. Furthermore, in contrast to other studies which took place in a laboratory sitting, we located the robot in an active rehabilitation setting where clinicians were responsible for its daily operation. One limitation of the study was the small sample size.

## Conclusions

Stroke is a leading cause of disability, particularly in the elderly, and robot-assisted techniques have important potential benefits. A prototype therapeutic robot system was built using a desk top industrial robot to rehabilitate the impaired upper limb of individuals at the subacute stage post stroke.

It can be concluded from the study results that robotic arm therapy alone, i.e., without additional physical therapy interventions tailored to the paretic arm, was as effective as standard physiotherapy treatment and more effective for improving the stage of motor impairment of the impaired arm. Our findings are promising for robotic systems to become a mainstay of inpatient stroke treatment. We anticipate that increased client participation, through independent practice and the various feedback effects of the robotic process, will further enhance recovery following a stroke.

## Competing interests

The authors declare that they have no competing interests.

## Authors' contributions

HA and CT developed the robotics system used in the study. CL and SB performed the daily therapy over the course of the clinical trial. BA performed statistical analysis on the results of the study. All authors drafted, read and approved the manuscript.

## References

[B1] Barreca S Wolf S Fasoli S Bohannon R Treatment interventions for the paretic upper limb of stroke survivors: A critical review Neurorehabil &Neural Repair 2003 17 220 226 10.1177/0888439003259415 14677218

[B2] Nakayama H Jorgensen HS Raaschou HO Olsen TS Compensation in recovery of upper extremity function after stroke: the Copenhagen Stroke Study Arch Phys Med Rehabil 1994 75 8 852 857 10.1016/0003-9993(94)90108-2 8053790

[B3] Fisher B Sullivan K Activity-dependent factors affecting poststroke functional outcomes Topics in Stroke Rehabil 2001 8 3 31 44 10.1310/B3JD-NML4-V1FB-5YHG 14523736

[B4] Schaechter JD Motor rehabilitation and brain plasticity after hemiparetic stroke Prog Neurobiol 2004 73 1 61 72 10.1016/j.pneurobio.2004.04.001 15193779

[B5] Biernaskie J Chemenko G Corbett D Efficacy of rehabilitative experience declines with time after focal ischemic brain injury J Neurosci 2004 24 5 1245 54 10.1523/JNEUROSCI.3834-03.2004 14762143 PMC6793570

[B6] Biernaskie J Corbett D Enriched rehabilitative training promotes improved forelimb motor function and enhanced dentritic grow after focal ischemic injury J Neurosci 2001 21 4 5272 80 11438602 10.1523/JNEUROSCI.21-14-05272.2001 PMC6762844

[B7] Consensus Panel on the Stroke Rehabilitation System Time is function Heart and Stroke Foundation of Ontario 2007

[B8] Mima T Sadato N Yazawa T Fukuyama H Yonekura Y Shibasaki H Brain structures related to active and passive finger movements in man Brain 1999 4 105 110 10.1093/brain/122.10.1989 10506099

[B9] Carel C Loubinoux I Thilman AF Neural substrate for the effects of passive training on sensorimtoor cortical representation: a study with functional magnetic resonance imaging in healthy subjects Cerebr Blood Flow Metab 2000 20 478 484 10.1097/00004647-200003000-00006 10724112

[B10] Woldag H Hummelsheim H Evidence-based physiotherapeutic concepts for improving arm and hand function in stroke patients: a review J Neurol 2002 249 518 528 10.1007/s004150200058 12021939

[B11] Van der LJ Snels I Beckerman H Lankhorst G wagenaar R Bouter L Exercise therapy for arm function in stroke patients: a systematic review of randomized controlled trials Clin Rehabil 2001 15 20 31 10.1191/026921501677557755 11237158

[B12] Teasel R Foley N Bhogal S Jutai J Speechley M Evidence-Based Review Stroke Rehabilitation, Retrieved http://08/1/09ebrsr.com/index_home.html

[B13] Kwakkel G Boudewijn J Kollem Krebs H Effects of robot-assisted therapy on upper limb recovery after stroke: A systematic review Neurorehabil & Repair 2008 22 2 111 121 10.1177/1545968307305457 PMC2730506 17876068

[B14] Fasoli SE Krebs HL Hogan N Robotic technology and stroke rehabilitation: Translating research into practice Top Stroke Rehabil 2004 11 11 19 10.1310/G8XB-VM23-1TK7-PWQU 15592986

[B15] Krebs HI Hogan NA Aisen ML Volpe BT "Robot-aided neuro-rehabilitation", IEEE Transactions on Rehabilitation Engineering " 1998 6 75 87 no.1 10.1109/86.662623 PMC2692541 9535526

[B16] Erlandson RF Application of robotic/mechatronic systems in special education, rehabilitation therapy, and vocational training: A paradigm shift" IEEE Transactions on Rehabilitation Engineering 1995 13 1 22 34

[B17] Dijkers MP de Bear PC Erlandson RF Kristy K Geer DM Nicholes A Patient and staff acceptance of robot technology in occupational therapy J Rehabil Research & Development 1991 28 2 33 44 10.1682/JRRD.1991.04.0033 2066869

[B18] Krebs HI Palazzolo JJ Dipietro L Ferraro M Krol J Rannekleiv K Volpe BT Hogan N Rehabilitation robotics: Performance-based progressive robot-assisted therapy Autonomous Robots 2003 15 7 20 10.1023/A:1024494031121

[B19] Krebs HI Mernoff S Fasoli SE Hughes R Stein J Hogan NA Comparison of functional and impairment-based robotic training in severe to moderate chronic stroke: A pilot study NeuroRehabil 2008 23 81 87 PMC4692808 18356591

[B20] Lo AC Guarino PD Richards LG Haselkorn JK Wittenberg GF Federman DG Robot-assisted therapy for long-term upper-limb impairment after stroke N Engl J Med 2010 362 1772 1783 10.1056/NEJMoa0911341 20400552 PMC5592692

[B21] Lum PS Burgar CG Shor PC Majmundar M Van der Loos M Robot-assisted movement training compared with conventional therapy techniques for the rehabilitation of upper-limb motor function after stroke Arch Phys Med Rehabil 2002 83 y663 673 10.1053/apmr.2001.33101 12098155

[B22] Lum PS Burgar CG Van der Loos M Shor PC Majmundar M Yap R MIME robotic device for upper-limb neurorehabilitation in subacute stroke subjects: A follow-up study Journal of Rehabilitation Research and Development 2006 43 5 631 42 10.1682/JRRD.2005.02.0044 17123204

[B23] Reinkensmeyer J Rian D Schmit Z Rymer Assessment of active and passive restraint during guided reaching after chronic brain injury Ann Biomedical Engineering 1999 27 6 952 959 10.1114/1.233 10625152

[B24] Coote S Murphy B Harwin W Stoke E The effect of the GENTLE/s Robot-mediated therapy system on arm function after stroke Clin Rehabil 2008 22 395 405 10.1177/0269215507085060 18441036

[B25] Hesse S Schmidt H Werner C Machine to support motor rehabilitation after stroke: 10 years of experience in Berlin J Rehabil Research & Devel 2006 43 671 678 10.1682/JRRD.2005.02.0052 17123207

[B26] Whitall J McCombe A Waller S Silver KH Macko RF Repetitive bilateral arm training with rhythmic auditory cueing improves motor function in chronic hemiparetic Stroke Stroke 2000 31 10 2390 2395 10.1161/01.STR.31.10.2390 11022069

[B27] Fazekas G Horvath M Troznai T Toth A Robot-mediated upper limb physiotherapy for patients with spastic hemiparesis: A preliminary study Journal of Rehabilitation Medicine 2007 39 7 580 2 10.2340/16501977-0087 17724559

[B28] Sivan M O'Connor R Makower S Levesley M Bhakta B Systematic review of outcome measures used in the evaluation of robot-assisted upper limb exercise in stroke J Rehabil Med 2011 43 181 189 10.2340/16501977-0674 21305232

[B29] Brewer BR McDowell SK Worthen-Chaudhari LC Poststroke upper extremity rehabilitation: a review of robotic systems and clinical results Top Stroke Rehabilitation 2007 4 6 22 44 10.1310/tsr1406-22 18174114

[B30] Khan L Zygman M Rymer W Reinkensmeyer D Robot-assisted reaching exercise promotes arm movement recovery in chronic hemiparetic stroke: A randomised controlled pilot study 6 Journal of NeuroEngineering & Rehabil 2006 3 2 1 13 16790067 10.1186/1743-0003-3-12 PMC1550245

[B31] Loureiro R Amirabdollahian F Topping M Driessen B Harwin W Upper limb robot mediated stroke therapy--GENTLE/s approach Autonomous Robots 2003 15 35 51 10.1023/A:1024436732030

[B32] Timmermans AA Seelen A Willmann RD Kingma H Technology-assisted training of arm-hand skills in stroke: concepts on reacquisition of motor control and therapist guidelines for rehabilitation technology design Journal of NeuroEngineering and rehab 2008 6 1 1 44 10.1186/1743-0003-6-1 PMC2647548 19154570

[B33] Mehrholz J Platz T Kugler J Pohl M Electromechanical and robot-assisted arm training for improving arm function and activities of daily living after stroke 2009 The Cochrane Collaboration 2009 4 Published by John Wiley & Sons, Ltd 1 34 10.1002/14651858.CD006876.pub2 18843735

[B34] Abdullah HA Tarry C Datta R Mittal GS Abderrahim M "A Dynamic Bio-mechanical Model to Assess and Monitor Robot-Assisted Therapy of Upper Limb Impairment" Journal of Rehabilitation Research and Development 2007 44 43 62 no 1 10.1682/JRRD.2006.03.0025 17551857

[B35] Barreca S Gowland C Stratford P Development of the Chedoke Arm and Hand Activity Inventory: Theoretical constructs item generation and selection Top Stroke Rehabil 2004 11 4 31 42 10.1310/JU8P-UVK6-68VW-CF3W 15592988

[B36] Barreca B Stratford P Lambert C Masters L Streiner D Test-retest reliability,validity and sensitivity of the Chedoke Arm and Hand Activity Inventory: A new measure of upper limb function for survivors of stroke Arch Phys Med &Rehabil 2005 86 1616 22 10.1016/j.apmr.2005.03.017 16084816

[B37] Barreca S Stratford P Masters L Lambert C Griffiths J McBay C Validation of three shortened versions of the Chedoke Arm and Hand Activity Inventory: The CAHAI-7, CAHAI-8 and CAHAI-9 Physiotherapy Canada 2006 58 1 9 10.3138/ptc.58.1.01

[B38] Barreca S Stratford P Masters L Lambert C Griffiths J Comparing 2 versions of the Chedoke Arm and Hand Activity Inventory with the Action Research Arm Test Phys Therapy 2006 86 2 245 253 16445338

[B39] Gowland C Stratford P Ward M Moreland J Torresin W Measuring physical impairment and disability with the Chedoke-McMaster Stroke Assessment Stroke 1993 24 58 63 10.1161/01.STR.24.1.58 8418551

[B40] SAS Institute Inc SAS/STAT User's Guide Cary, NC: SAS Institute, Inc

[B41] Snedecor G Cochran W Statistical methods 1989 8 Ames: Iowa State University Press

